# The role of tumor-associated macrophages in breast cancer progression

**DOI:** 10.3892/ijo.2013.1938

**Published:** 2013-05-14

**Authors:** ELIAS OBEID, RITA NANDA, YANG-XIN FU, OLUFUNMILAYO I. OLOPADE

**Affiliations:** 1 Section of Hematology Medical and Oncology, Department of Medicine, University of Chicago, Chicago, IL 60637, USA; 2 Department of Pathology and Committee on Immunology, University of Chicago, Chicago, IL 60637, USA; 3 Center for Clinical Cancer Genetics and Global Health, University of Chicago, Chicago, IL 60637, USA

**Keywords:** breast cancer, tumor-associated macrophges, hypoxia, tumor microenvironment, toll-like receptors, extracellular matrix, vascular endothelial growth factor, angiogenesis

## Abstract

It is well established that the tumor microenvironment plays a major role in the aggressive behavior of malignant solid tumors. Among cell types associated with tumor microenvironment, tumor-associated macrophages (TAMs) are the most influential for tumor progression. Breast cancer is characterized by having a large population of TAMs, and experimental models have exposed multiple mechanisms by which TAMs interact with and influence the surrounding tumor cells. The process of metastasis involves tumor cells gaining access to the tissue outside the immediate tumor environment and invading the confining extracellular matrix (ECM). Supporting this process, TAMs secrete proangiogenic factors such as VEGF to build a network of vessels that provide nutrition for tumor cells, but also function as channels of transport into the ECM. Additionally, TAMs release factors to decrease the local pro-inflammatory antitumor response, suppressing it and providing a means of escape of the tumor cells. Similarly, hypoxia in the tumor microenvironment stimulates macrophages to further produce VEGF and suppress the T-cell immune responses, thus, enhancing the evasion of tumor cells and ultimately metastasis. Given the multiple roles of TAMS in breast cancer progression and metastasis, therapies targeting these cells are in development and demonstrate promising results.

## Contents

1. Introduction
2. Tumor-associated macrophages
3. TAMs and hypoxia
4. TAMs and angiogenesis
5. TAMs and the tumor matrix
6. TAMs and toll-like receptors
7. Clinical applications
8. Conclusion

## 1. Introduction

Tumors of epithelial cell origin grow in a complex and dynamic stroma consisting of various cell types, matrix proteins and soluble factors. This microenvironment provides all the necessary stimuli for tumor viability, growth, and invasiveness. Essential components of this microenvironment are inflammatory cells and the pro-inflammatory cytokines they produce. The role of inflammation in cancer progression was first described by Virchow in 1867 (1). Recent epidemiologic evidence suggests that inflammation resulting from pathogen stimulation could be a promoter of tumorigenesis by driving the immune response through different inflammatory cells that are recruited to and reside in the tumor microenvironment (2). Processes that enhance the aggressiveness of tumor behavior include angiogenesis, hypoxia, interaction between cell membrane receptors and tumor microenvironment cytokines and basement membrane degradation.

The process of metastasis and invasiveness of tumor cells is complex and determines clinical outcomes of cancer patients. Many studies have identified a link between this process and some of the cellular elements in the tumor microenvironment. Cells in the tumor microenvironment secrete a wide array of products that modulate the behavior of tumor cells in a tumor-enhancing and tumor-suppressing fashion. Of these cells, macrophages have gained a considerable interest in recent years. Macrophages are the most abundant inflammatory cells in the solid tumor microenvironment, and in this review, we examined how macrophages enhance the invasiveness of breast cancer.

## 2. Tumor-associated macrophages

Tumor-associated macrophages (TAMs) have been described in many solid tumors, including breast cancer. They make up a big proportion of the cellular elements in different tumors. In breast cancer, macrophages can constitute as much as 50% of the cell mass (3). Multiple clinical studies have shown a correlation between breast cancer prognosis and the number of macrophages in the tumor mass. In a recent report by Bingle *et al*, a meta-analysis showed that an increased macrophage density was associated with poor prognosis in more than 80% of breast cancer cases (4).

### Origin of TAMs

Macrophages are derived from the mononuclear phagocyte system (5). Progenitor cells in the bone marrow can give rise to two types of cells: neutrophils and macrophages. The macrophage pathway is committed to produce monoblasts through serial divisions of promonoblasts from the colony forming unit, granulocyte-macrophage (CFU-GM). Peripheral blood monocytes, derived from bone marrow progenitor cells that give rise to monoblasts and promonocytes, eventually leave the blood stream and enter the tissue where they differentiate into macrophages (6). In the tissues, they have different roles based on the tissue in which they reside. Leek and Harris (5) described macrophages as the ‘Swiss army knife’ of the immune system. However, when they are activated appropriately, macrophages have specific and distinct functions. Many signals influencing macrophages have been identified and include hypoxia, lipids, and cytokines (e.g., CCL2, CCL3 and CSF-1), TNF-α, INF-γ and MIF (7). Macrophage migration into tissues is not haphazard; rather, this process is specifically orchestrated by various chemoattractants. Among the chemoattractants, CCL2 (formally referred to as monocyte chemoattractant prorein-1, MCP-1) is suggested to be important in tumor progression. Fujimoto *et al* showed recently that stromal CCL2 is associated with recruitment of macrophages to the breast cancer stroma and plays a role in relapse-free survival (8). Macrophage migration inhibitory factor (MIF) is another chemoattractant involved in tumor progression. MIF, an upstream regulator of host immunity, constitutes an important link between chronic inflammation and cancer. By interacting with CXCR2 and CXCR4, MIF recruits leukocytes and activates cellular inflammatory responses (9). Its levels were found to be elevated in different cancers, particularly prostate and breast cancer. Of particular interest, intratumoral MIF levels were inversely related to nodal status, and this determined an interaction between MIF and intratumoral interleukins (10). A series of experiments with breast cancer cell lines and macrophage cell lines showed that MIF was a major gene product that was upregulated when breast cancer cells were cocultured with macrophages. MIF secreted by tumor cells increased the macrophage metalloproteinase production rates and facilitated tumor cell invasion (11).

### Macrophage polarization

After entering peripheral tissues, the pattern of macrophage activation depends on the surrounding microenvironment, which accounts for the heterogeneity among macrophage populations and their plasticity. Typically, macrophages are divided into two main groups, the M1 and M2 macrophages.

**Figure 1.**
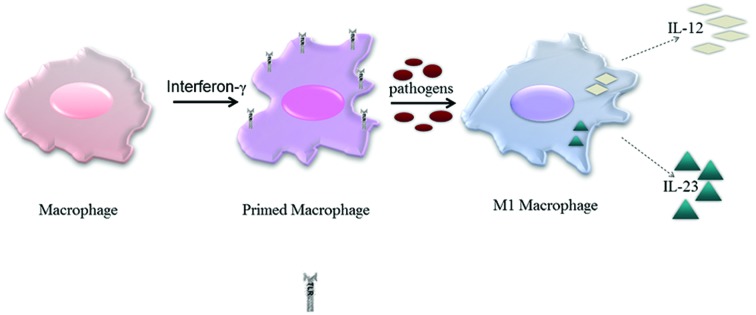
Schematic representation of the polarization of macrophages into M1 macrophages (classically activated macrophages). INF-γ primes macrophages causing an upregulation of toll-like receptors (TLRs) so that in the presence of pathogens (red discs) or pathogen products (e.g., lipopolysaccharide), those primed macrophages acquire an M1 phenotype releasing cytokines, particularly IL-12 and IL-23.

M1 macrophages (also known as ‘classically’ activated macrophages) are an integral cellular component of the immune system. These cells play an important role in protection against intracellular pathogens and cancer cells by virtue of the type I immune response they activate. They are ‘primed’ by the cytokine INF-γ for activation either by tumor necrosis factor-α (TNF-α) or (and more importantly) by activation of toll-like receptors (TLRs) via exposure to microbes or microbial products such as bacterial LPS (12). Once activated, M1 macrophages start secreting pro-inflammatory cytokines such as interferons and interleukins. M1 macrophages particularly secrete high levels of IL-12 and in humans they also secrete high levels of IL-23 (13). M1 macrophages also function as antigen-presenting cells; a function that is regarded as a potentially antitumor function (14). These cells have an amplified capacity to kill intracellular pathogens by generating toxic oxygen species and by activating the inducible NO synthase (iNOS) gene to produce nitric oxide (NO). Therefore, M1 macrophages can defend the host from tumor cells and infections (14–16) (Fig. 1).

**Figure 2.**
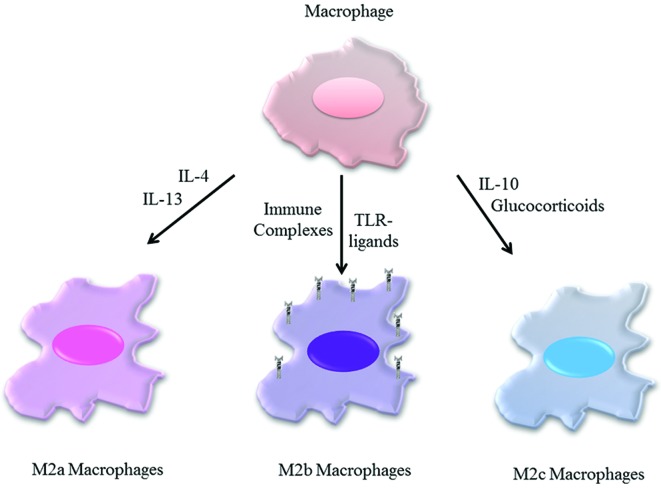
Schematic representation of the polarization of the M2 macrophages by their respective stimuli. M2a macrophages are macrophages activated by IL-4 and IL-13. In the presence of immune complexes and TLR-ligands, macrophages are polarized into an M2b phenotype. Both M2a and M2b phenotypes have anti-inflammatory function and pro-tumorigenic properties. In the presence of glucocorticoids and IL-10, macrophages are polarized into M2c macrophages, and they have an immunosuppressive activity.

The M2 group of macrophages is referred to as tumor-associated macrophages as they generally promote tumor growth and metastasis (17). They are also referred to as ‘alternatively activated’ macrophages (M2). Unlike M1 macrophages that are activated by INF-γ, M2 macrophages are activated by other cytokines or by immune complexes. This activation induces a T-helper 2 type of response and as such, they are appropriately called M2 macrophages (reflecting this Th2-type response). However, it has recently been suggested that this M1/M2 dichotomous assignment of macrophages might be too simple and does not convey the different functions and activation patterns of M2 macrophages. An alternative classification was proposed by Mantovani *et al* whereby M2 macrophages are divided into three sub-populations depending on the particular stimulus that activates them (17). M2a (alternative) macrophages are induced by exposure to IL-4 and IL-13 while M2b macrophages are induced by toll-like receptor ligands (e.g., LPS) and immune complexes. Both the M2a and M2b groups promote the Th2 immune response, by secreting Th2 type cytokines and by recruiting Th2 cells (17). The M2c macrophage subgroup represents macrophages that are activated by IL-10 and glucocorticoid hormones. In general, an M2c response is responsible for suppressing inflammation which is an immunologically a Th-1 type of response (Fig. 2).

Macrophages seem to be directed by the tumor microenvironment and in turn depending on the surrounding milieu will acquire a trophic property as opposed to the original immune role they had (18). It is clear that different stimuli polarize macrophages into different phenotypes. *In vitro* models have shown that macrophages can change their polarization pattern according to the different stimuli (19). This suggests their dynamic interaction with the tumor microenvironment changing it constantly favoring tumor cell invasion and subsequent metastasis.

## 3. TAMs and hypoxia

### Hypoxia and macrophage recruitment

Lower oxygen concentrations in tumors have been verified and reproduced in many studies. In solid tumors the chaotic disorganized array of vascularization with blind ended vessels and leaky endothelial lining leads to a low oxygen tension (hypoxic condition) in such tumors (20). Breast cancer, like other solid malignancies, is hypoxic (21), making the oxygen supply insufficient for the rapidly growing tumor. In addition to the areas of low oxygen supply, necrotic areas in a tumor bed develop an acute drop in oxygen with ensuing cell death. This hypoxic environment within the tumor is one factor that influences macrophage function as debris in this area attracts macrophages (22). The research done by Turner *et al* showed that once attracted, macrophages are entrapped in necrotic tumor areas (23). One explanation for this phenomenon is that upregulation of mitogen-activated protein kinase phosphatase (MKP-1) dephosphorylates chemoattractant receptors for VEGF and CCL2 (VEGFR and CCR2, respectively), thus aborting their chemotactic response in TAMs (24,25). This accumulation of TAMs in hypoxic areas has been associated with aggressive tumor behavior in breast cancer (26).

### Hypoxia upregulates VEGF expression in TAMs

Hypoxia is known to regulate gene expression in both cancer and inflammatory cells within the tumor microenvironment. Hypoxia-induced gene expression in macrophages is in part due to the upregulation of the transcription factors HIF-1α and HIF-2α (27). HIF-1α is a marker for hypoxia and in breast cancer it has been linked to aggressive malignant behavior (28). Indeed, HIF-1α-dependent genes are associated with an increased patient mortality in some cancers (29). So far VEGF remains the most notable gene upregulated by HIF-1α and HIF-2α secondary to hypoxic stress in tumor microenvironment. Lewis *et al* showed that TAMs express VEGF almost exclusively in perinecrotic and poorly vascularized areas in breast cancer (30). In breast cancer, the overexpression of HIF-2α in TAMs was related to increased tumor vascularity from the upregulation of VEGF expression by the effect of HIF-2α (31).

### Hypoxia promotes immune evasion by TAMs

Hypoxia-induced HIF-1α also promotes tumor progression by promoting immune evasion. Under hypoxic conditions TAMs secrete more immunosuppressive cytokines such as IL-10, which inhibits immune effector T-cells (32). In fact, using a murine model of breast cancer Doedens *et al* recently reported that tumor growth is decreased with deletion of HIF1α in macrophages, despite normal levels of VEGF and tumor vascularization. In this model, TAMs suppressed the antitumor activity of tumor-infiltrating T cells (33). The loss of HIF-1α in myeloid cells reversed the hypoxia-induced suppression of T-cell activation resulting in tumor progression. Overall, this study demonstrated that T cells of the adaptive immune response are suppressed by the myeloid derived cells of innate immune system in a HIF-1α-dependent and hypoxia-induced fashion.

### Hypoxic TAMs promote metastatic behavior of tumor cells

Promoting tumor metastatic behavior is another mechanism whereby macrophages in hypoxic breast cancers increase tumor aggressiveness. Macrophage migratory inhibitory factor (MIF) is one of the gene products increased by HIF-1α upregulation in hypoxic conditions. It was found that MIF is released by hypoxic macrophages (34). Recently, Oda *et al* showed that the effect of MIF on HIF-1α is p53-dependent process and that MIF stabilizes HIF-1α protein under hypoxic conditions (35). MIF's stimulatory effect on the release of matrix metalloproteinases [e.g., MIF-induced increased expression of MMP-9 (36)], which degrade the basement membranes in tumors, opens the gates for tumor cells to escape and initiate a nidus of metastatic foci.

Together the above studies reveal that hypoxic conditions drive accumulation of TAMs in necrotic and poorly vascularized areas of breast cancer. Once in the tumor, the microenvironment influences macrophages to enhance the aggressive behavior of tumors. These hypoxia-mobilized pro-tumoral activities of TAMs may serve as potential targets for breast cancer therapy. By interfering at various levels prior to metastatic and invasive tumor activity the progression induced by macrophages may be reversed. For example, since radiation therapy is known to induce hypoxia in tumor beds, blocking VEGF or CCL2 could potentially stop the recruitment of macrophages to these hypoxic tumor areas and slow or stop tumor progression.

## 4. TAMs and angiogenesis

### TAMs secrete VEGF, promoting tumor angiogenesis

Focal TAM infiltration was shown to have an effect on prognosis in breast cancer. It is well known now that TAMs can affect tumors in different ways, and one of the major pathways is by stimulating angiogenesis (26). TAMs in breast carcinomas express numerous tumor-promoting factors, the most prominent of which is the proangiogenic vascular endothelial growth factor (VEGF) (30). In a series of breast cancer cases, Leek *et al* found that there was a positive correlation between high mean vessel density and increased macrophage index in the tumor, as well as an inverse relationship between macrophage counts and relapse-free and overall survival (26). Subsequently, they showed that there is an association between vascular endothelial growth factor (VEGF) and macrophage infiltration (37) increasing the evidence for a proangiogenic role for TAMs in breast cancer. Therefore, it became reasonable to think that the effect on prognosis which macrophages have is in part related to their proangiogenic effect. Indeed, breast cancer spheroids containing macrophages induce overexpression of VEGF and an increase in the number of vessels when injected in dorsal skin folds of nude mice (38). Further evidence comes from a study that showed the level of post-surgery circulating serum VEGF levels were directly dependent on macrophage infiltration of the primary tumor as well as the presence of somatic mutation of *p53* gene (39). However, this relationship needs to be further investigated to validate its effect on the metastatic activity of macrophage rich tumors.

Using an elegant transgenic mouse model, Lin *et al* demonstrated that inhibition of macrophage maturation and infiltration into tumors delayed the formation of a high-density vessel network. In this model, genetically deleting colony stimulating factor-1 (CSF-1), a key chemoattractant of macrophages, makes PyMT mice susceptible to mammary cancer. However, restoring CSF-1 showed that tumor-associated macrophages play a key role in fostering tumor angiogenesis which is an essential step in the tumor progression to malignancy (40). They later elaborated that genetically restoring VEGF to the tumor microenvironment in these macrophage-depleted animals restores tumor progression through macrophage-produced VEGF stimulating tumor angiogenesis (41).

### TAMs selectively have an angiogenic gene signature

Gene expression profiling has been utilized to understand the different macrophage populations in breast cancer. In an experimental mouse model, Ojalvo *et al* were able to use a flow cytometry technique to isolate a specific TAM population from late stage mammary tumors that was previously shown to be associated with an invasive mammary carcinoma. For comparison, they isolated a similar macrophage population from the spleens of mice that did not have mammary tumors. Gene expression signatures for 460 genes from both populations were studied. They found that the transcript abundance was differentially regulated between the two populations. Macrophage functions such as suppression of immune activation, matrix remodeling and secretion of tumor angiogenesis mediators were higher in the TAM population from the mammary tumor (42). This indicates that a TAM gene expression signature in mouse tumors could be used to assess expression of TAMs in human breast cancer. These data suggest TAMs may regulate tumor angiogenesis and therefore support further investigation into the role of other transcriptional mediators of TAM function within the tumor microenvironment (42). In addition, gene expression profiling may be a promising methodology to explain the different populations of TAMs and their potential functions. It also may be helpful in differentiating tumors by their aggressive potential.

From the highlighted studies, it is clear that macrophages contribute to tumor growth, invasion, and metastasis through an angiogenic pathway. Clinically, macrophages could be a potential target of anticancer therapy, where possibly a treatment would limit metastatic spread and thus increase overall survival. In the angiogenesis pathway, clinical trials have already been studying the targeting of proangiogenic factors such as VEGF. Therefore, an indirect targeted therapy against macrophages or their products is already underway, yet they are not selectively aimed at macrophages which could provide excellent opportunities for more targeted therapies (43). Moreover, detailed gene expression profiling of tumor-associated macrophages will eventually be used to determine what pathway(s) to target for each particular tumor.

## 5. TAMs and the tumor matrix

Tumor cells and inflammatory cells of the tumor microenvironment are surrounded by extracellular matrix (ECM). The growth of tumor cells locally and their ability to invade and metastasize depends partially on how they can get around this matrix as well as by how they can modulate it to help their evasion.

### TAMs and matrix degrading enzymes

TAMs produce several enzymes which can degrade the extracellular matrix (ECM). Such enzymes include several metalloproteinases (e.g., MMP-2 and MMP-9) as well as urokinase-type plasminogen activator (uPA) that degrade the ECM (44). Dissolution of the ECM leads to cleavages through which tumor cells can evade and metastasize. Clinically, uPA has been under investigation in breast cancer. In one study, it was shown that those patients whose uPA activity was elevated in breast cancer tissue had a significantly shorter disease-free interval compared to those with low levels of activity (45). Recently uPA and plasminogen activator inhibitor type 1 (PAI-1) have received greater interest in breast cancer research and treatment and are possible novel tumor biological markers. In fact, uPA is possibly a potent independent prognostic factor in breast cancer (46). Currently, the NNBC-3 trial is investigating treatment of node-negative early stage breast cancer classified as high or low risk based on uPA and its inhibitor PAI-1 (47). Therefore, this draws attention to the importance of the ECM and macrophages in breast cancer progression and even targeted therapy.

### Macrophages and fibrillary collagen in ECM

Fibrillary collagen is an important substrate of the extracellular matrix. Using multi-photon microscopy Wyckoff *et al* revealed these structures and found that surrounding the tumor there is a dense ring of fibrillary collagen (48). Moreover, tumor cells were seen moving alone the collagen fibers ten times faster than they do through the stroma. Tumor cells move directly towards vessels along the collagen fibers. Ingman *et al*, found that depleting macrophages during the process of mammary tumor development in a mouse model, reduced the extent of collagen I synthesis and restoration of macrophages resulted in correcting this defect (49). Further studies are needed to understand how exactly macrophage relate to this collagen fibrillogenesis and tumor invasiveness.

## 6. TAMs and toll-like receptors

Toll-like receptors (TLRs) are a family of transmembrane receptors that recognize conserved molecular patterns of microbial origins. TLRs play an important role in tissue repair and their role in cancer progression is being more appreciated (50). In breast cancer, there is an increased evidence for an overexpression of certain TLRs in the more aggressive breast cancer subtypes (51). It is well known that TLRs are expressed on mononuclear inflammatory cells (52). The relationship of TLRs and macrophages in breast cancer is in its infancy. The data are still limited but based on some other tumor models, there seems to be a role for macrophages and TLRs in breast cancer. In a Lewis lung carcinoma cell line model, Kim *et al* showed that cancer cells can activate macrophages leading to production of IL-6 and TNF-α via TLR2 and TLR6 activation (53). In a mouse model of inflammation-induced liver cancer by diethylnitrosamine (DEN), debris from hepatocytes activated macrophages via the MyD88 pathway. Interestingly, gender difference favoring the development of this tumor in female mice was identified. Naugler *et al* (54) showed that the genetic deletion of IL-6 decreased the incidence of liver cancer and the presence of estradiol (E2) conferred protection from developing liver cancer even in IL-6-deficient mice after IL-6 was exogenously restored (IL-6-deficient mice with exogenously restored IL-6 developed liver cancer in male mice and in ovariectomized female mice). Although this model is based on a hepatic carcinoma *in vivo* model, this interesting finding, of TLR activation pathway seemed to be protective in the presence of estrogen receptor (particularly ERα) which raises the question of whether there is a protective role for estradiol in women with breast cancer that would be lost in chemical/physiological menopause, that is mediated through TLRs and tumor-associated macrophages. If this is proven then targeting this pathway may confer protection from disease progression. Indeed, ERα may be related to the downregulation of certain TLRs in breast cancer (55).

On the other hand, some studies showed a positive role for TLR signaling in stimulating a regulatory immune response in tumor microenvironment. The high mobility group box 1 protein (HMGB1) is released from apoptotic cells and activates TLR 4 on dendritic cells, stimulating them to function as cross-presenters of antigens (MHC I), thus stimulating T-cell responses (56,57).

Mutation of TLR4 gene in humans was found to have an increased frequency of metastasis in breast cancer patients (56). The counterpart mutation in TLR4 in mouse models showed a decreased response to both chemotherapy and radiation therapy for mammary cancer (56). In a cell line model, hypoxic stress was found to increase the TLR4 expression in macrophages in a HIF1α-regulated manner (58). These results suggest that TLR4 activity in immune cells of the tumor microenvironment and the innate immunity are involved in the inflammatory process that is hypoxia-driven. However, further studies are required to evaluate the role of different TLRs and macrophage activation in breast cancer.

## 7. Clinical applications

Research studying the malignant potential of transformed epithelial cells, such as mammary cells, showed that these cells acquire a malignant potential; however, this alone is not sufficient to render them able to grow and sustain tumor growth unless in a permissive environment (59). The breast cancer microenvironment is rich with inflammatory cells of which TAMs play a significant role. Augmented by tumor hypoxia, TAMs may potentially affect the clinical course of the tumor by potentiating an invasion permissive environment. Additionally, TAMs secrete proangiogenic factors such as VEGF stimulating the growth of tumor vasculature to sustain the growing malignant mass (18). They also secrete extracellular degrading proteases (e.g., uAP) allowing for tumor cells to escape and metastasize. Moreover, TAMs suppress the proinflammatory response that is initiated against tumor cells allowing them to grow and divide.

Clinically, the role of TAMs is gaining importance. For example, in a study by Allavena *et al*, trabectedin (Yondelis^®^) which is a novel antitumor agent of marine origin was found to affect differentiated macrophages as well as freshly isolated TAMs at effective therapeutic doses (60). The selective cytotoxic effect of Yondelis on TAMs significantly reduced their production of IL-6 and CCL2 (a major monocyte chemoattractant recruiting them to tumor tissue). This compound can be seen as the prototype of a new class of breast cancer therapies since it targets inflammation-driven tumors with high TAM component.

As we discussed, tumor-associated macrophages play an important role in enhancing the vasculature of tumors, by secreting the vascular endothelial growth factor (VEGF). Anti-VEGF antibodies such as bevacizumab, can stop this process of vasculogenesis that a tumor mass needs in order to sustain its viability. Additionally, therapies that target VEGF receptor 2 may decrease macrophage infiltration in the tumor microenvironment (61). This can be a potential therapeutic target, as not only would it work by its anti-angiogenic activity, but also by decreasing the macrophage recruitment which is the source of VEGF secretion and further angiogenesis. The addition of a VEGF inhibitor to chemotherapy has been shown to increase progression-free survival for metastatic breast cancer, but not overall survival (62,63). Clearly, there appears to be a subset of patients who benefit from the addition of anti-angiogenesis therapy to chemotherapy, and macrophage density in such tumors may be the guide to the subset of patients who benefit most from this therapy.

The bisphosphonate zoledronic acid (Zometa^®^) is of particular interest in the treatment of women with breast cancer. Not only does it prevent demineralization of bones from hormone manipulation therapy and decreases skeletal related events in metastatic breast cancer, but it also increases disease-free survival in young women when given early in the treatment have been reported (64). Many studies on this amino-bisphosphonate showed that its anticancer activity is related to inhibiting angiogenesis and decreasing the recruitment and differentiation of monocytes into TAMs (65). In one laboratory model, the amino-bisphosphonate zoledronic acid was found to suppress MMP-9 expression by infiltrating macrophages and inhibit its ECM degrading activity (66). These data suggest zoledronic acid could be one of the new medications targeting TAMs by an ‘unconventional MMP-9 inhibition’.

Further work in immunotherapy aimed at activating macrophages toward the M1 lineage and therefore, depleting or reducing other macrophage populations (M2) would be another way of targeting macrophages in the clinical setting. Activation of TLR-9 by ligands such as CpG-oligodeoxynucleotides has been used in an early clinical trial of lymphoma and non-small cell lung cancer. In an *in vitro* model, the addition of TLR-9 CpG oligonucleotide and an IL-10 receptor antibody induced a shift in the residence and recruited tumor-infiltrating macrophages from the M2 into the M1 type in mouse mammary tumors (67). Therefore, although the therapeutic targeting of TAMs is still in early stages, it is nevertheless promising.

## 8. Conclusion

The tumor microenvironment is rich in inflammatory cells and their products. Current research done indicates that among these cells, TAMs play a key role in breast cancer progression and invasiveness. Recruited by tumor cells, TAMs are preferentially molded to serve the need of the cancer cells in a pro-tumoral function instead of an anticancer role and as such, potentiate their malignant behavior. More research needs to be done in the field to understand the intricate relationship between macrophages and breast cancer in order to translate the findings into meaningful clinical trials of therapeutics with TAMs as a target.
